# Synthesis and Preliminary Evaluation of the Antimicrobial Activity of Selected 3-Benzofurancarboxylic Acid Derivatives

**DOI:** 10.3390/molecules15074737

**Published:** 2010-07-06

**Authors:** Jerzy Kossakowski, Mariola Krawiecka, Bożena Kuran, Joanna Stefańska, Irena Wolska

**Affiliations:** 1 Department of Medical Chemistry, Medical University of Warsaw, 3 Oczki Str., 02-007 Warsaw, Poland; E-Mails: jerzy.kossakowski@wum.edu.pl (J.K.); bozena.kuran@wum.edu.pl (B.K.); 2 Department of Pharmaceutical Microbiology, Medical University of Warsaw, 3 Oczki Str., 02-007 Warsaw, Poland; E-Mail: joanna.stefanska@wum.edu.pl (J.S.); 3 Department of Crystallography, Faculty of Chemistry, Adam Mickiewicz University, 6 Grunwaldzka Str., 60-780 Poznań, Poland; E-Mail: iwolska@amu.edu.pl (I.W.)

**Keywords:** 3-benzofurancarboxylic acid, antimicrobial activity, antifungal activity, X-ray diffraction

## Abstract

Halogen derivatives of selected 3-benzofurancarboxylic acids were prepared using 6-acetyl-5-hydroxy-2-methyl-3-benzofuranocarboxylic acid as starting material. ^1^H-NMR spectra were obtained for all of the synthesized structures, and for compound **VI**, an X-ray crystal structure was also obtained. All derivatives were tested for antimicrobial activity against a selection of Gram-positive cocci, Gram-negative rods and yeasts. Three compounds, **III**, **IV**, and **VI**, showed antimicrobial activity against Gram-positive bacteria (MIC 50 to 200 μg/mL). Compounds **VI **and **III** exhibited antifungal activity against the *Candida* strains *C. albicans* and *C. parapsilosis* (MIC – 100 μg/mL).

## 1. Introduction

**Figure 1:**
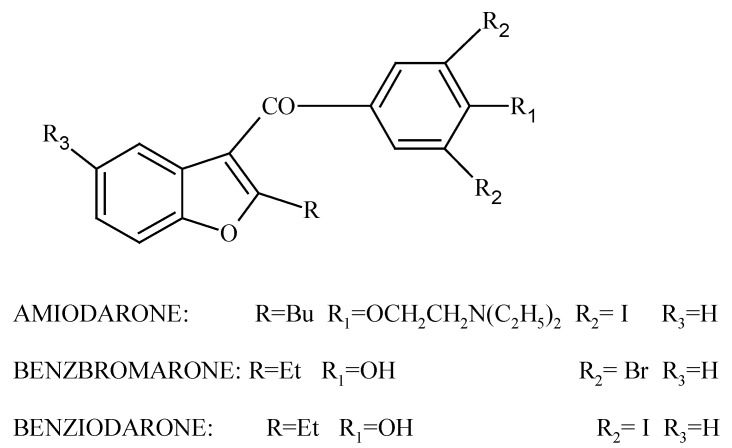
The structures of amiodarone, benzbromarone and benzidarone.

It is well known that many of heterocyclic compounds containing oxygen display important biological properties such as antiarrhytmic, spasmolitic, antiviral, anticancer, antifungal and anti-inflammatory activity [1,2,3,4,5,6,7]. This group of compounds includes the furobenzopyranone, benzofuran, and benzopyranone systems. Good examples of the above mentioned are the drugs amiodarone, benziodarone and benzbromarone (Figure 1). 

**Figure 2:**
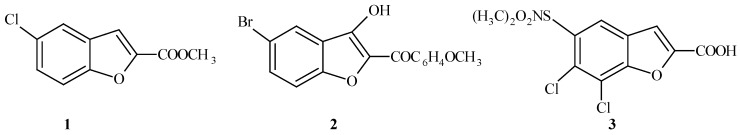
The structures of halo derivatives of benzofurans: methyl 5-chloro-1-benzofuran-2-carboxylate (**1**); 5-bromo-3-hydroxy-1-benzofuran-2-yl)(4-methoxyphenyl)methanone (**2**); 6,7-dichloro-5-[(*Z*)-[methoxy(oxido)-λ^5^-azanylidene](methyl)-λ^4^-sulfanyl]-1-benzo-furan-2-carboxylic acid (**3**).

Amiodarone is used clinically as an antiarrhytmic agent causing alternations in calcium homeostasis, and cell death in yeasts and it also possesses antifungal activity [1,8,9,10,11]. Benziodarone is a vasodilator [12], and benzbromarone is effective in lowering uric acid levels, as well as reducing the number of acute gout attacks in patients for whom other treatments are ineffective [13,14]. Although the last two compounds were withdrawn from the market because of their serious side effects, amiodarone is still used. Due to the interesting biological activity of these compounds, it seemed worthwhile to search for new compounds with similar structures in order to identify potentially less toxic compounds, much safer for health and the environment. Other examples of compounds with related structures showing biological activity (Figure 2) can be found in the literature. Compound **1**, for example, shows high affinity for adrenergic receptors and possesses antidepressive activity [16]. Derivative **2** inhibits absorption of biogenic amines [15] and **3** decreases blood pressure [17].

**Figure 3:**
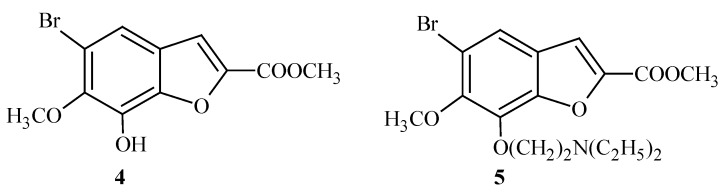
The structures of methyl 5-bromo-7-hydroxy-6-methoxy-1-benzofuran-2-carboxylate (**4**); methyl 5-bromo-7-[2-(diethylamino)ethoxy]-6-methoxy-1-benzofuran-2-carboxylate (**5**).

For many years we have been involved in research in the field of synthesis of new biologically active benzofurans. A large number of compounds synthesized by our team contain halogens in their structure and some of them have shown biological activity [19,20,21], e.g. compounds **4 **and **5 **(Figure 3).

**Figure 4:**
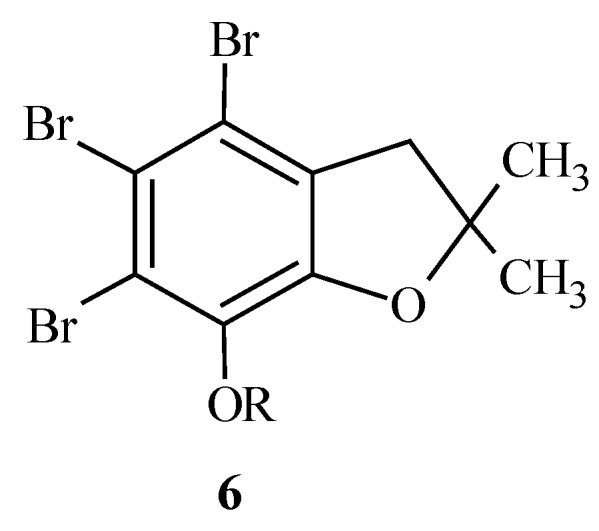
The general structure **6** of 1-(3-amino-2-hydroxypropyl) derivatives of 4,5,6-tribromo-2,3-dihydro-2,2-dimethyl-7-benzofuranol.

These two compounds showed significant cytotoxic activity against human cancer cell lines. Additionally, derivative **5** demonstrated antifungal activity. Some 1-(3-amino-2-hydroxypropyl) derivatives of 4,5,6-tribromo-2,3-dihydro-2,2-dimethyl-7-benzofuranol (Figure 4) are effective against Gram-positive bacteria and fungi [20,21]. Our research shows that brominated compounds display lower cytotoxity than the corresponding precursor compounds before bromination. [20,21].

**Figure 5:**
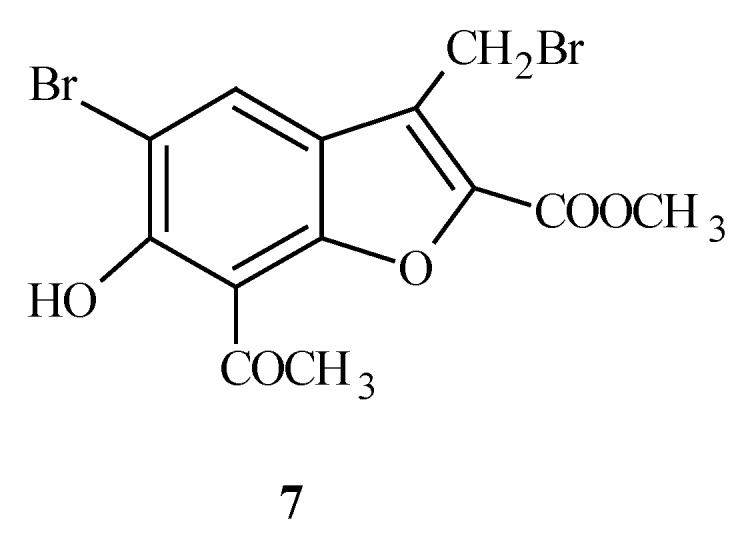
The structure of methyl 7-acetyl-5-bromo-3-(bromomethyl)-6-hydroxy-1-benzofuran-2-carboxylate (**7**).

Compound **7** (Figure 5) and its analogues are the topic of a patent application [22]. Compounds of this invention exhibit synergistic antifungal activity in combination with the antifungal compound amiodarone. They are further useful as antifungal agents for the prevention and/or treatment of fungal infections in plants.

**Scheme 1:**
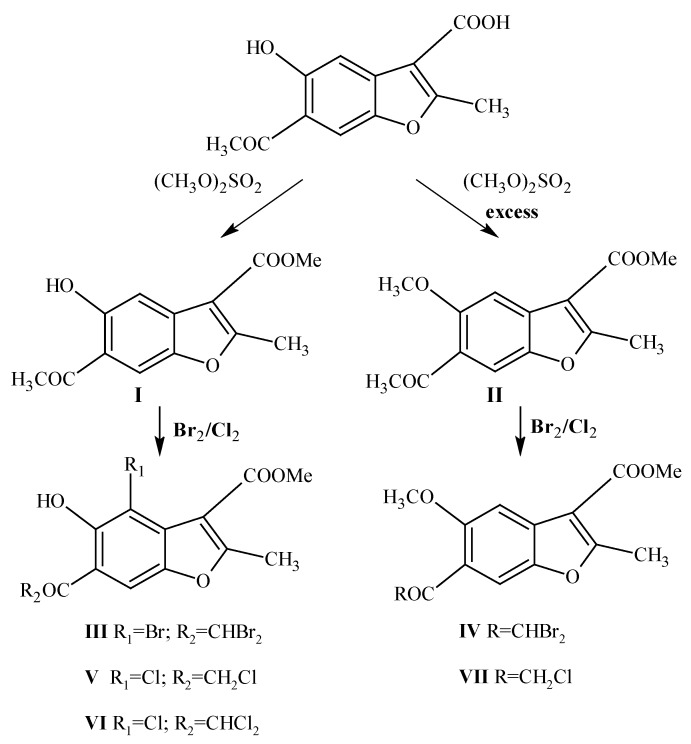
Method of preparation of compounds **I**–**VII**.

In light of this information we have decided to continue our research, and thus design and synthesize some new compounds that might show antimicrobial activity (Scheme 1). 

## 2. Results and Discussion

### 2.1. Chemistry

The chosen starting material was 6-acetyl-5-hydroxy-2-methyl-1-benzofuran-3-carboxylic acid. Compounds **I** [18,19] and **II** were obtained by mono- or dimethylation of the starting material with dimethyl sulphate, respectively. Next, the corresponding bromo and/or chloro derivatives were synthesized. As a result, the benzofuran ring of compound **I** was substituted on C4 and the acetyl group by halogen and we thus obtained methyl 4-bromo-6-(dibromoacetyl)-5-hydroxy-2-methyl-1-benzofuran-3-carboxylate (**III**), methyl 4-chloro-6-(chloroacetyl)-5-hydroxy-2-methyl-1-benzofuran-3-carboxylate (**V**) and methyl 4-chloro-6-(dichloroacetyl)-5-hydroxy-2-methyl-1-benzofuran-3-carboxylate (**VI**). In the case of compound **II**, only acetyl group halogenations were observed (**IV**, **VII**). ^1^H-NMR spectra were obtained for all of the synthesized structures, and for compound **VI** an X-ray crystal structure was obtained too.

### 2.2. NMR spectra

Analysis of the ^1^H-NMR spectra for the obtained derivatives **III**, **V** and **VI** shows no acetyl group signals, whereas for 6-acetyl-5-hydroxy-2-methyl-3-benzofuranocarboxylic acid we observe a singlet signal for this group for with a chemical shift value of 2.78 ppm. On the other hand, we observed additional signals at 4.74 ppm for compound **V**, at 6.73 ppm for compound **VI**, and at 6.72 ppm for compound **III**, indicating that hydrogens in the acetyl group were substituted by chlorine or bromine. Additionally, for these compounds we do not observe any hydrogen signal for C4, which suggests that it was substituted by chlorine or bromine, too. We obtained similar ^1^H-NMR spectra for compounds **IV,** **VII**.

### 2.3. X-Ray structure analysis

**Figure 6:**
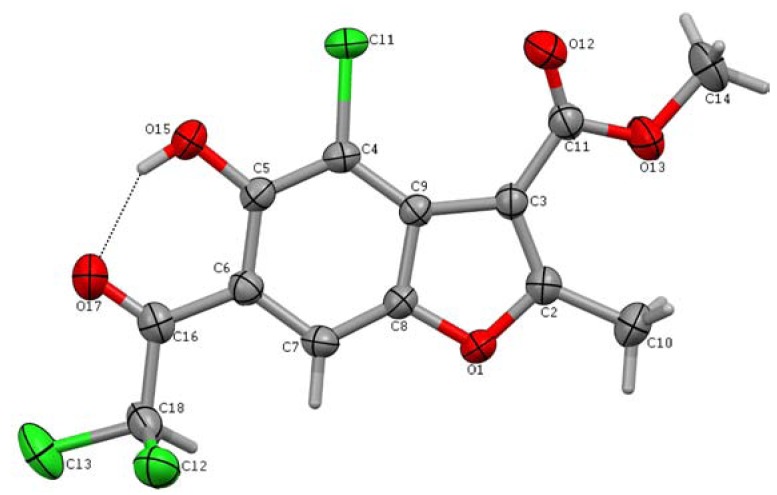
A view of the molecule of **VI**.

In order to confirm the structure of **VI**, the molecular and crystal structure in the solid state was analyzed by single crystal X-ray diffraction. Suitable crystals could only be obtained only for this derivative. A view of the molecular structure together with the atomic numbering scheme is shown in Figure 6 (the drawings were performed with the Mercury program [23]). 

The results indicate that compound **VI** crystallizes in the monoclinic space group P 2_1_/c with one ordered molecule in the asymmetric unit. Selected bond lengths, bond angles and torsion angles are listed in Table 1. 

**Table 1:** Selected bond lengths [Å] and angles [deg] and selected torsional angles [deg] for **VI**.

O1-C2	1.354(2)
O1-C8	1.371(2)
C5-O15	1.346(2)
C5-C6	1.429(3)
C6-C16	1.463(3)
C2-O1-C8	107.1(1)
C3-C11-O13	110.4(2)
C6-C16-O17	123.3(2)
C9-C3-C2-C10	-178.5(2)
C3-C9-C4-Cl1	-0.1(3)
C7-C6-C5-O15	-177.3(2)
C6-C16-C18-Cl2	81.9(2)
C6-C16-C18-Cl3	-157.7(2)

The geometry of the benzofuran ring is comparable to that found in other benzofuran derivatives [19,24,25,26]. Only the C5-C6 bond length in the benzene seems rather longer due to the substituent at C6 indicating a π-electron delocalization within this fragment of the molecule. The benzofuran moiety is essentially planar with a maximum deviation of 0.017(2)Å for C6. The methoxycarbonyl group is planar to within 0.007Å and makes an angle of 18.65(6)^o^ with plane of the benzofuran system. The C10, O15, Cl1 atoms are almost coplanar with the benzofuran fragment (the appropriate torsion angles are given in Table 1) and the C16, O17 and C18 atoms are found to be only marginally out of the plane of the two-ring framework [max. deviation of 0.172(3)Å for O17]. Only halogen atoms, Cl2 and Cl3, are considerably out of the mentioned plane (see Table 1). Strong intramolecular hydrogen bonding is present between O15 and O17 atoms (see Figure 6 and Table 2). The angle between the best planes of the benzofuran and the C5/O15/H15/O17/C16/C6 moiety is only 4.37(4)^o^. Additionally the C11=O12···Cl1-C4 halogen bond [3.018(2)Å] stabilizes the conformation of the molecule.

**Figure 7:**
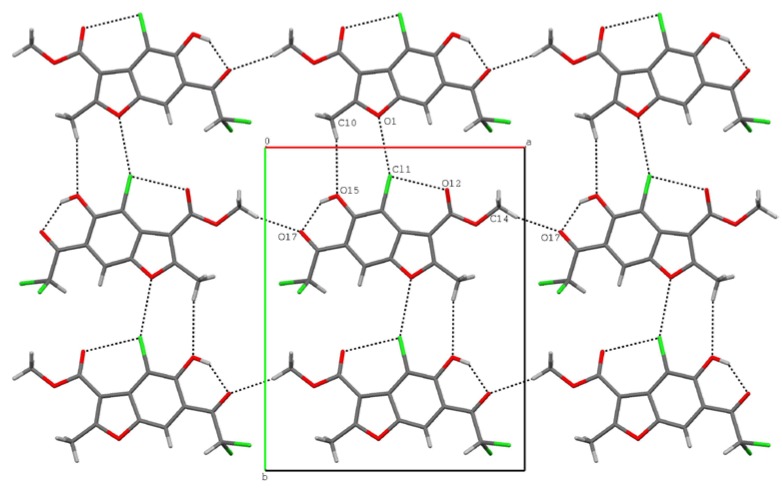
The interconnections within a layer for **VI**.

In the crystal of **VI**, the packing of the molecules is determined by intermolecular C-H···O hydrogen bonds, Cl···O, C···O interactions and stacking forces. The molecules are linked by C14-H14A···O17 hydrogen bonds forming chains along the *a* axis, then adjacent chains are connect *via* C10-H10A···O15 and O1···Cl1 interactions to form layers parallel to the (001) plane (Figure 7).

Cohesion between sheets results in Cl···O, C···O interactions and stacking forces. The geometric parameters of all intra- and intermolecular interactions are given in Table 2. 

**Table 2:** Intra- and intermolecular interactions in crystals of **VI **(Å, deg).

D-H···A	D-H	H···A	D···A	<( D-H···A)
O15-H15···O17	0.82	1.86	2.580(2)	146
C10-H10A···O15^i^	0.96	2.82	3.758(3)	165
C14-H14A···O17^ii^	0.96	2.66	3.470(3)	142
O12···Cl1			3.018(2)	
O1···Cl1^i^			2.995(1)	
C2···O12^iii^			3.122(3)	
Cl3···O15^iii^			3.457(2)	
C16···O15^iii^			3.238(3)	

Symmetry codes: (i) -x+1, y+1/2, -z+1/2; (ii) x+1, y, z; (iii) x, -y+1/2, z+1/2.

### 2.4. Antimicrobial activity

All the synthesized 6-acetyl-5-hydroxy-2-methyl-3-benzofuranocarboxylic acid derivatives were tested for antimicrobial activity. The microorganisms used in this study included Gram-positive cocci, Gram-negative rods and yeasts. Compounds **III**, **IV**, **VI** were active against Gram-positive cocci: *Staphylococcus aureus* NCTC 4163, *Staphylococcus aureus* ATCC 25923, *Staphylococcus aureus* ATCC 6538, *Staphylococcus aureus* ATCC 29213, *Staphylococcus epidermidis* ATCC 12228, *Bacillus subtilis* ATCC 6633, *Bacillus cereus* ATCC 11778, *Micrococcus luteus* ATCC 9341, *Micrococcus luteus* ATCC 10240. Compounds **III**, **VI **showed antifungal activity against *Candida albicans* ATCC 10231, *Candida albicans* ATCC 90028 and *Candida parapsilosis* ATCC 22019. The results are summarized in Table 3.

**Table 3:** Antimicrobial activity of derivatives **III**, **IV**, **VI**: diameter of the growth inhibition zone.

	Growth inhibition zones in mm (MIC values)				
Compound strain	III	IV	VI	Ciprofloxacin	Fluconazole
*S. aureus* NCTC 4163	12 (200)	12 (50)	12 (100)	26 (0.5)	nt
*S. aureus* ATCC 25923	12 (200)	13 (50)	12 (200)	26 (0.5)	nt
*S. aureus* ATCC 6538	12 (200)	14 (50)	12 (100)	28 (0.5)	nt
*S. aureus* ATCC 29213	12 (200)	13 (50)	12 (100)	22 (0.5)	nt
*S. epidermidis* ATCC 12228	12 (100)	14 (50)	13 (100)	30 (0.5)	nt
*B. subtilis* ATCC 6633	12 (100)	13 (50)	13 (100)	40 (<0.125)	nt
*B. cereus* ATCC 11778	12 (100)	15 (50)	12 (100)	20 (1)	nt
*E. hirae* ATCC 10541	- (> 400)	- (200)	- (400)	- (4)	nt
*M. luteus* ATCC 9341	12 (400)	15 (100)	12 (200)	22 (2)	nt
*M. luteus* ATCC 10240	12 (100)	15 (50)	12 (100)	24 (1)	nt
*E. coli* ATCC 10538	na	na	na	34 (<0.125)	nt
*E. coli* ATCC 25922	na	na	na	35 (<0.125)	nt
*E. coli* NCTC 8196	na	na	na	35 (<0.125)	nt
*P. vulgaris* NCTC 4635	na	na	na	36 (<0.125)	nt
*P. aeruginosa* ATCC 15442	na	na	na	25 (0.5)	nt
*P. aeruginosa* NCTC 6749	na	na	na	26 (0.5)	nt
*P. aeruginosa* ATCC 27853	na	na	na	23 (1)	nt
*B. bronchiseptica* ATCC 4617	na	na	na	31 (1)	nt
*C. albicans* ATCC 10231	12 (100)	na	13 (100)	nt	22 (1)
*C. albicans* ATCC 90028	12 (100)	na	12 (100)	nt	32 (1)
*C. parapsilosis* ATCC 22019	13 (100)	na	14 (100)	nt	22 (2)

na - no activity in disc diffusion test; - denotes lack of a growth inhibition zone; nt – not tested.

Unsubstituted esters **I**, **II** showed no microbiological activity. The change of microbiological activity appears when a halogen is introduced into a benzofuran structure. Derivatives in which an acetyl group hydrogen was substituted by one halogen (**V**, **VII**) were inactive, in contrast to derivatives in which two acetyl group hydrogens were substituted by halogens (**III**, **IV**, **VI**). These compounds were active against Gram-positive cocci. Additionally, derivatives containing a halogen in the aromatic ring (**III**, **VI**) showed antifungal activity (Table 3).

## 3. Experimental

### 3.1. General

Melting points were determined in a capillary in Electrothermal 9100 apparatus and are uncorrected. The proton nuclear magnetic resonance spectra (^1^H-NMR) were recorded in CDCl_3_ on a Varian UNITY-plus 300 spectrometer operating at 300 MHz. Chemical shift values are expressed in ppm (parts per million) in relation to tetramethylsilane as an internal standard. Mass spectral ESI (Electrospray Ionization) measurements were carried out on a Mariner PE Biosystems instrument with TOF detector. The spectra were obtained in the positive ion mode with a declustering potential 140–300 V. Elemental analyses were recorded with CHN model 2400 Perkin-Elmer. Chromatographic columns were filled with Merck Kieselgel 0.05–0.2 mm reinst (70–325 mesh ASTM) silica gel. Reactions were monitored by TLC on silica gel (plates with fluorescent indicator 254 nm, layer thickness 0.2 mm, Kieselgel G., Merck), using chloroform-methanol 98:2 and 95:5 as eluents.

*Methyl 6-acetyl-5-hydroxy-2-methyl-1-benzofuran-3-carboxylate* (**I**). A mixture of 6-acetyl-5-hydroxy-2-methyl-1-benzofuran-3-carboxylic acid (0.02 mole), K_2_CO_3_ (0.02 mole) and (CH_3_O)_2_SO_2 _(0.02 mole) in acetone (30 mL) was refluxed for 5 h. When the reaction was complete, the mixture was filtered and the solvent was evaporated. The residue was purified by column chromatography on silica gel, eluent: chloroform. Yield: 87%; m.p. 170–171 °C; ^1^H-NMR δ (ppm): 12.17 (s, 1H, OH), 7.76 (s, 1H, C7-H), 7.46 (s, 1H, C4-H), 3.93 (s, 3H, COOCH_3_), 2.78 (s, 3H, COCH_3_), 2.67 (s, 3H, CH_3_); Anal. Calc. for C_13_H_12_O_5_: 62.90% C; 4.87% H; found: 62.87% C, 4.87% H; ESI MS m/z: 247.1, 248.8 (100%).

*Methyl 6-acetyl-5-methoxy-2-methyl-1-benzofuran-3-carboxylate* (**II**). A mixture of 6-acetyl-5-hydroxy-2-methyl-1-benzofuran-3-carboxylic acid (0.02 mole), K_2_CO_3_ (0.02 mole) and excess (CH_3_O)_2_SO_2 _(0.06 mole) in acetone (30 mL) was refluxed for 10 h. When the reaction was complete, the boiling mixture was filtered and the solvent was evaporated. The residue was purified by column chromatography on silica gel, eluent: chloroform. Yield: 78%; m.p. 118–119 °C;^ 1^H-NMR δ (ppm): 7.83 (s, 1H, C7-H), 7.48 (s, 1H, C4-H), 3.87 (s, 3H, COOCH_3_), 3.95 (s, 3H, OCH_3_), 2.76 (s, 3H, COCH_3_), 2.65 (s, 3H, CH_3_); Anal*.* Calc. for C_14_H_14_O_5_: 64.12% C; 5.38% H; found 64.19% C; 5.39% H.

### 3.2 General procedure for synthesis of bromo derivatives of esters ***I*** and ***II***

The appropriate ester **I** or **II** (0.02 mole) was dissolved in CHCl_3_ (20 mL) then a solution of bromine (0.04 mole) in CHCl_3_ (10 mL) was added dropwise with stirring for 0.5 h. Stirring was continued for 8 h at room temperature. When the reaction was complete the solvent was evaporated. The residue was purified by column chromatography on silica gel, eluent: chloroform.

*Methyl 4-bromo-6-(dibromoacetyl)-5-hydroxy-2-methyl-1-benzofuran-3-carboxylate* (**III**). Yield: 77%; m.p. 149–150 °C; ^1^H-NMR δ (ppm): 11.98(s, 1H, OH), 8.00 (s, 1H, C7-H), 6.72 (s, 1H, COCHBr_2_), 3.95 (s, 3H, COOCH_3_), 2.63 (s, 3H, CH_3_); Anal. Calc. for C_13_H_9_O_5_Br_3_: 32.20% C; 1.87% H; found 32.38% C; 2.09% H; ESI MS m/z: 506.8:508.7 [M+Na]^+^ (100%).

*Methyl 6-(dibromoacetyl)-5-methoxy-2-methyl-1-benzofuran-3-carboxylate* (**IV**). Yield: 78%; m.p. 135–138 °C; ^1^H-NMR δ (ppm): 7.95 (s, 1H, C7-H), 7.54 (s, 1H, C4-H), 7.22 (s, 1H, COCHBr_2_), 4.05 (s, 3H, COOCH_3_), 3.98 (s, 3H, OCH_3_), 2.80 (s, 3H, CH_3_); Anal*.* Calc. for C_14_H_12_O_5_Br_2_: 40.03% C; 2.88% H; found 39.94% C; 2.77% H.

### 3.3. General procedure for synthesis of chloro derivatives of esters ***I*** and ***II***

The appropriate ester **I** or **II** (0.02 mole) was dissolved in CHCl_3_ (20 mL). Next chlorine_,_ obtained in the reaction of KMnO_4_ with concentrated HCl, was passed through the solution. When the reaction was complete the solvent was evaporated. The residue was purified by column chromatography on silica gel, eluents: chloroform and chloroform/methanol 100:0.5.

*Methyl 4-chloro-6-(chloroacetyl)-5-hydroxy-2-methyl-1-benzofuran-3-carboxylate* (**V**). Yield: 23%; m.p. 187–188 °C; ^1^H-NMR δ (ppm): 12.11 (s, 1H, OH), 7.74 (s, 1H, C7-H), 4.74 (s, 2H, COCH_2_Cl), 3.96 (s, 3H, COOCH_3_), 2.68 (s, 3H, CH_3_);; Anal. Calc. for C_13_H_10_O_5_Cl_2_: 49.24% C; 3.18% H; found 49.14% C; 3.24% H; ESI MS m/z: 339.6:341[M+Na]^+^ (100%).

*Methyl 4-chloro-6-(dichloroacetyl)-5-hydroxy-2-methyl-1-benzofuran-3-carboxylate* (**VI**). Yield: 36%; m.p. 171–172 °C;^ 1^H-NMR δ (ppm): 11.70 (s, 1H, OH), 7.98 (s, 1H, C7-H), 6.73 (s, 1H, COCHCl_2_), 3.96 (s, 3H, COOCH_3_), 2.68 (s, 3H, CH_3_); Anal. Calc. for C_13_H_9_O_5_Cl_3_: 44.41% C; 2.58% H; found 44.60% C; 2.71% H; ESI MS m/z: 350.8:352.6 [M+H]^+^ (100%).

*Methyl 6-(chloroacetyl)-5-methoxy-2-methyl-1-benzofuran-3-carboxylate* (**VII**). Yield: 54%; m.p. 130–131 °C; ^1^H-NMR δ (ppm): 7.69 (s, 1H, C7-H), 7.19 (s, 1H, C4-H), 4.75 (s, 2H, COCH_2_Cl), 3.88 (m, 6H, COOCH_3_, OCH_3_), 2.60 (s, 3H, CH_3_); Anal. Calc. for C_14_H_13_O_5_Cl*2H_2_O: 50.53% C; 3.90% H; found 50.60% C; 3.90% H.

### 3.4. Crystallography

Crystals of **VI** suitable for X-ray analysis were grown from acetic acid solution by slow evaporation. All details of the measurements, crystal data and structure refinement are given in Table 4. The data were collected on an Oxford Diffraction KM4CCD diffractometer [27] at 293 K, using graphite-monochromated *MoK_α_* radiation. The unit cell parameters were determined by least-squares treatment of setting angles of highest-intensity reflections chosen from the whole experiment. Intensity data were corrected for the Lorentz and polarization effects [28]. The structure was solved by direct methods by use the SHELXS97 program [29] and refined by the full-matrix least-squares method with the SHELXL97 program [30]. Two reflections were excluded from the reflection file due to their large (|F_o_|^2^-|F_c_|^2^) differences. The function Σw(|F_o_|^2^-|F_c_|^2^)^2^ was minimized with w^‑1 ^= [σ^2^(F_o_)^2^+(0.0474P)^2^+0.6944P], where P = (F_o_^2^+2F_c_^2^)/3. All non-hydrogen atoms were refined with anisotropic thermal parameters. The coordinates of the hydrogen atoms were calculated in idealized positions and refined as a riding model with their thermal parameters calculated as 1.2 (1.5 for methyl group) times U_eq_ of the respective carrier carbon atom. An empirical extinction correction was also applied according to the formula F_c_’ = kF_c_[1+(0.001χF_c_^2^λ^3^/sin2θ)]^-1/4^ [30], and the extinction coefficient χ was equal to 0.008(1). The deposition number CCDC 700768 contains the supplementary crystallographic data of compound **VI** for this paper. These data can be obtained free of charge via www.ccdc.cam.ac.uk/data_request/cif, or by emailing data_request@ccdc.cam.ac.uk, or by contacting The Cambridge Crystallographic Data Centre, 12, Union Road, Cambridge CB2 1EZ, UK; fax: +44 1223 336033. 

**Table 4:** Crystal data, data collection and structure refinement for compound **VI**.

Compound	**VI**
Empirical formula	C_13_H_9_O_5_Cl_3_
Formula weight	351.55
*T* (K)	293(2)
Wavelength (Å)	0.71073
Crystal system, space group	monoclinic, *P* 2_1_/c
Unit cell dimensions	
*a* (Å)	13.0544(3)
*b* (Å)	15.7053(3)
*c* (Å)	7.0108(2)
*β* (^o^)	104.897(3)
Volume (Å^3^)	1389.07(6)
*Z*, *D_x_*(Mg/m^3^)	4, 1.681
*μ* (mm^-1^)	0.677
*F*(000)	712
*θ* range for data collection (^o^)	2.07 – 25.00
*hkl* range	-15 ≤ h ( 15
	-18 ( k ( 18
	-8 ( l ( 8
Reflections:	
collected	13369
unique (Rint)	2409(0.019)
observed (I > 2((I))	2003
Data / restraints / parameters	2409 / 0 / 191
Absorption correction	multi-scan
Goodness-of-fit on *F^2^*	1.055
*R*(*F*) (*I* > 2*σ*(*I*))	0.0293
*wR*(*F^2^*) (all data)	0.0895
Max/min. Δ*ρ* (e/ Å^ 3^)	0.315 / -0.237

### 3.5. Microbiology

The antimicrobial activity of compounds was tested against a series of microorganisms obtained from the collection of the Department of Pharmaceutical Microbiology, Medical University of Warsaw (Poland), including: Gram-positive cocci: *Staphylococcus aureus* NCTC 4163, *Staphylococcus aureus* ATCC 25923, *Staphylococcus aureus* ATCC 6538, *Staphylococcus aureus* ATCC 29213, *Staphylococcus epidermidis* ATCC 12228, *Bacillus subtilis* ATCC 6633, *Bacillus cereus* ATCC 11778, *Enterococcus hirae* ATCC 10541, *Micrococcus luteus* ATCC 9341, *Micrococcus luteus* ATCC 10240; Gram-negative rods: *Escherichia coli* ATCC 10538, *Escherichia coli* ATCC 25922*,Escherichia coli* NCTC 8196, *Proteus vulgaris* NCTC 4635, *Pseudomonas aeruginosa* ATCC 15442, *Pseudomonas aeruginosa* NCTC 6749, *Pseudomonas aeruginosa* ATCC 27863, *Bordetella bronchiseptica* ATCC 4617 and yeasts: *Candida albicans* ATCC 10231, *Candida albicans* ATCC 90028, *Candida parapsilosis* ATCC 22019. 

Antimicrobial activity was examined by the disc-diffusion method and the MIC method under standard conditions using Mueller-Hinton II agar medium (Becton Dickinson) for bacteria or RPMI agar medium (Sigma) with 2% glucose for fungi, according to CLSI guidelines [31,32]. For the disc diffusion assay sterile filter paper discs (9 mm diameter, Whatman No 3 chromatography paper) were wetted with tested compound solutions (in DMSO) to load 400 μg of a given compound per disc. For MIC determinations concentrations of tested compounds in solid medium ranged from 6.25 to 400 μg/mL. The final inoculum, of all microorganisms were 10^4^ CFU mL^-1^ (colony forming units per mL), except the final inoculum for *E. hirae* ATCC 10541, which was 10^5^ CFU mL^-1^. The results were read after 18 h (for bacteria) or 24 h (for fungi) of incubation at 35 °C. The results are summarized in Table 3. 

## 4. Conclusions

This study describes the synthesis and preliminary microbiological investigations of some 3-benzofurancarboxylic acid derivatives. Seven derivatives were examined. It was noticed that compounds not possessing halogen in their structure did not show any activity during the experiments, whereas compounds possessing halogens in their structure showed such activity. Halogens were introduced to alkyl substituent as well as to the aromatic ring. Derivatives in which the halogen, without the regard for the kind, was introduced in the aromatic ring, showed better activity, as these compounds displayed both antimicrobial and antifungal activity. Basing on these findings, as well as on results from our earlier investigations, we can confirm that the search for biologically active compounds in the area of halogen derivatives of benzofurans remains an interesting field from the biological point of view.
